# Stable Expression of Basic Fibroblast Growth Factor in Chloroplasts of Tobacco

**DOI:** 10.3390/ijms17010019

**Published:** 2015-12-23

**Authors:** Yun-Peng Wang, Zheng-Yi Wei, Xiao-Fang Zhong, Chun-Jing Lin, Yu-Hong Cai, Jian Ma, Yu-Ying Zhang, Yan-Zhi Liu, Shao-Chen Xing

**Affiliations:** 1 Jilin Provincial Key Laboratory of Agricultural Biotechnology, Agro-Biotechnology Research Institute, Jilin Academy of Agricultural Sciences, No. 1363, Shengtai st., Changchun 130033, China; wangypbio@163.com (Y.-P.W.); weizy@cjaas.com (Z.-Y.W.); zhongxf@cjaas.com (X.-F.Z.); lincj@cjaas.com (C.-J.L.); yuying0609@126.com (Y.-Y.Z.); liuyz_g@126.com (Y.-Z.L.); 2 Institute of Agricultural Quality Standard and Testing Technology, Jilin Academy of Agricultural Sciences, No. 1363, Shengtai st., Changchun 130033, China; yhcai64@163.com; 3 Faculty of Agronomy, Jilin Agricultural University, No. 2888, Xincheng st., Changchun 130118, China; majian19790106@163.com; 4 College of Biological Sciences, China Agricultural University, No. 2 West Yuanmingyuan Road, Beijing 100094, China

**Keywords:** tobacco, chloroplast, genetic transformation, basic fibroblast growth factor, green fluorescent protein

## Abstract

Basic fibroblast growth factor (bFGF) is a multifunctional factor in acceleration of cell proliferation, differentiation and transference, and therefore widely used in clinical applications. In this study, expression vector pWX-Nt03 harboring a codon-optimized *bFGF* gene was constructed and introduced into the tobacco chloroplasts by particle bombardment. After four rounds of selection, *bFGF* was proved to integrate into the chloroplast genome of regenerated plants and two of four transgenic plants were confirmed to be homoplastomic by PCR and Southern hybridization. ELISA assay indicated that *bFGF* represented approximately 0.1% of total soluble protein in the leaves of transplastomic tobacco plants. This is the first report of *bFGF* expression via chloroplast transformation in model plant, providing an additional option for the production of chloroplast-produced therapeutic proteins.

## 1. Introduction

Fibroblast growth factors (FGF) are a large family of heparin-binding protein mitogens, with potential therapeutic utilities due to their broad target cells specificity. Twenty three different FGFs have been described thus far [1]. One FGF, basic fibroblast growth factor (bFGF or FGF2), a single-subunit protein with a molecular weight of ~17 kDa and a pI of 9.6, was first cloned from the bovine brain and pituitary in 1978 [2].

bFGF acts to accelerate cellular proliferation, differentiation and transference [3,4,5]. Recombinant bFGF is highly desirable in the marketing due to its critical role in the growth of blood vessels, wound healing, tissue regeneration and nerve system [6,7,8] and many attempts have been made to meet the great demand over the past few decades. Currently bFGF is produced via genetic engineering technology in several systems, from conventional microbial expression systems like *Escherichia coli* (*E. coli*) [9,10], *Bacillus subtilis* [11] and yeast [12,13], to insect cells [14]. More importantly, two main crops, soybean and rice, were reported to express functional recombinant bFGF specifically in seeds by *Agrobacterium*-mediated transformation [15,16]. However, the insolubility and biological activity of target proteins would likely be an intractable obstacle for microbial [17] and plant-produced [16] platform to lag the further application when high level of protein expression was achieved. Plant-based production of therapeutic proteins or antigens can be cost-effective, as, unlike microbial systems, it does not require sterile laboratories or production equipment [18,19]. But, thus far the efficiency of plant nuclear transformation with many genes remains low, producing low protein yields complicated by gene silencing and unstable inheritance [20]. Moreover, pollen-mediated transgene flow has drawn significant attention due to its potential impact on biodiversity [21]. Chloroplast transformation will be likely to overcome those disadvantages due to the features of higher expression, generally soluble proteins and mostly maternal inheritance, although some challenges such as protein degradation, pleiotropic effects, time-consuming homoplasmic selection are still the main hurdles for most of plants and need to be addressed [22,23]. Since tobacco was firstly engineered via chloroplast transformation, plastid transformation has drawn more attention, achieving high-level expression, gene-stacking, no gene silencing, and even facilitating successful transfer of whole metabolic pathways to plastids [24]. 

In this study, we engineered tobacco plants to express bFGF via plastid transformation. To our knowledge, this is the first report of bFGF expressed via plastid transformation and therefore provides a novel alternative of bFGF expression. 

## 2. Results

### 2.1. Construction of Expression Vector and Functional Verification

**Figure 1:**
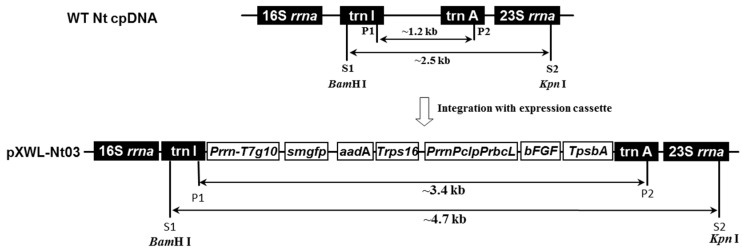
Construction of chloroplast transformation vector pXWL-Nt03. pXWL-Nt03 vector flanked with chloroplast regions of *16S-trnI* and *trnA-23S* comprised a synthetic expression cassette containing the *smgfp* reporter gene and *aadA* selectable marker gene. These two genes were jointly driven by the Prrn-T7g10 promoter and the terminator was 3’ untranslated regions (UTR) rps16 (T*rps16*). The target basic fibroblast growth factor (*bFGF*) gene was driven by the fusion promoter *PrrnPclpPrbcL* previously described [26] and *psbA* 3’ UTR as terminator (T*psbA*). P1/P2 and S1/S2 indicated the primers used for PCR and digestion sites for Southern blot analysis, respectively. A ~3.4 kb fragment was amplified for transplastomic plants, but a ~1.2 kb fragment in wild-type tobacco chloroplast DNA (WT Nt cpDNA) without expression cassette. Similarly, total DNA was digested by *BamH* I and *Kpn* I for Southern blot analysis. A ~4.7 kb signal was hybridized for transplastomic plants, but only a ~2.5 kb for untransformed plants.

An expression vector named pWXL-Nt03 for tobacco chloroplast transformation was constructed based on previously published protocol used in our laboratory [25] with two modifications (Figure 1). The homologous fragment from alfalfa was replaced with one from the tobacco plastid genome, and the codon-optimized *bFGF* gene was inserted into the vector multiple cloning site of the vector. The vector was verified by sequencing.

The chloroplast originated from a prokaryote and thus contains prokaryotic protein synthesis machinery. The specific expression vector for chloroplast transformation was therefore generally transferred into prokaryotic system like *E. coli* to test its genetic construction and function [27]. After the MACH 1, an *E. coli* strain, was transformed with this vector following the heat shock protocol, the overnight-cultured cells were collected by centrifugation. The pellet was observed to appear green in color, indicating the successful expression of green fluorescent protein (GFP) in transformed bacterial cells. This observation suggested that the other genes within the same expression cassette will play their roles in prokaryotic-like chloroplast expression system.

### 2.2. Tobacco Chloroplast Transformation and Homoplastomic Selection

**Figure 2:**
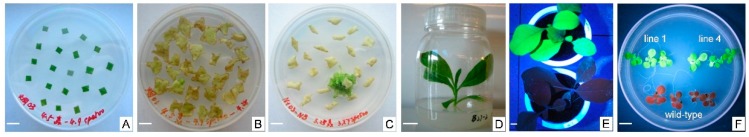
Procedure for homoplastomic selection after chloroplast transformation of tobacco. (**A**) Bombarded leaves were cut into pieces for selection; (**B**) Resistant shoots appear while most leave pieces bleached on antibiotic medium; (**C**) Surviving antibiotic resistant shoots; (**D**) Regenerated plantlet after first round of selection; (**E**) GFP expression in T_0_ transplastomic tobacco with green color and chlorophyll autofluorescence with red color under ultraviolet (UV) lamp; (**F**) GFP expression in T_3_ homoplastomic tobacco seedlings, line 1 and line 4, and wild-type control under UV lamp. Scale bars = 1 cm.

Young leaves were placed on the Murashige and Skoog (MS) medium with adaxial side down, cultured for 4 h in the dark and bombarded. The bombarded leaves were cultured in the dark at 26 °C for 3 days before being cut into 5 mm × 5 mm pieces and transferred into the selection media containing 500 mg/L of spectinomycin. Selection media was replaced every 3 weeks. Forty-three resistant transformants were regenerated in selection media, and integration of the expression cassette was verified by PCR. Young leaves from PCR-positive shoots were cut into pieces and placed abaxial side down in selective media and cultured for two to three additional regeneration cycles to obtain homoplastomic plants (Figure 2). Seeds were harvested from homoplastomic tobacco plants and sowed in soil until to T_3_ generation.

### 2.3. Molecular Characterization of T_0_ Transplastomic Plants

**Figure 3:**
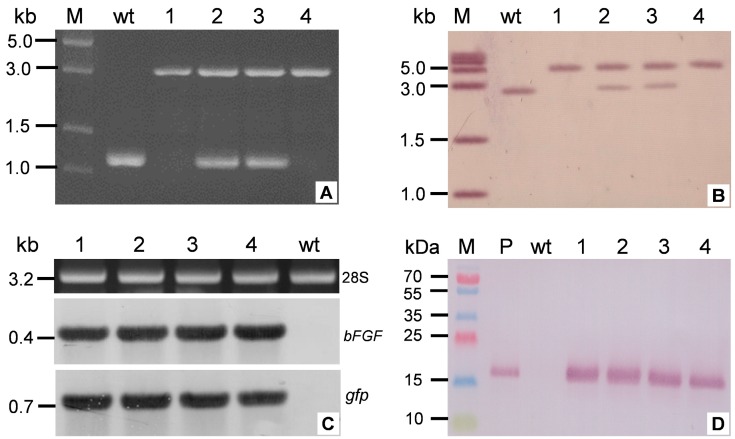
Molecular testing of T_0_ transplastomic plants. (**A**) PCR analysis of integration of the expression cassette into transgenic plants. M, DNA molecular marker; Lanes 1–4: T_0_ putative transgenic plants; (**B**) Southern blot analysis. M, DNA molecular marker; Lanes 1–4: the leaves of T_0_ putative transgenic plants. The single 4.7 kb band in lanes 1 to 4 indicated homoplastomic status due to the insertion of foreign expression cassette, whereas lanes 2 and 3 with an additional ~2.5 kb band, amplified from wild-type plants, indicating heteroplastomic status; (**C**) Northern blot analysis for *bFGF* (middle) and *gfp* (bottom) mRNAs. The ethidium bromide-stained gel of 28S band of total RNA is shown in the top as a loading quantitative control. wt is untransformed tobacco as negative control; (**D**) Western blot for determining the expression of bFGF in T_0_ transplastomic plants. Expression of bFGF (17 kDa) is observed in the leaves of four transgenic plants (lanes 1–4), but absent in the leaves of a wild-type plant (wt). Commercial bFGF protein (P) served as positive control. M is protein marker.

After four rounds of selection, antibiotic resistant shoots were obtained and transferred to new media without phytohormone for rooting. Integration of the foreign expression cassette into the plastid genome of transformants was verified by PCR. As shown in Figure 3A, two putative transgenic plants were homoplastomic with the single band of ~3.4 kb, but an additional ~1.2 kb band in lanes 2 and 3 indicated the heteroplastomic status of other two plants. Both hetero-/homoplastomic plants displayed normal phenotypes compared with wild-type plants under greenhouse condition.

Total DNA was isolated from the leaves of T_0_ transgenic and wild-type plants and integration of the *bFGF* gene into the tobacco plastid genome was confirmed by Southern blot. As indicated in Figure 3B, the single ~4.7 kb band in lane 1 and 4 implied homoplastomic status, whereas lanes 2 and 3 displayed an additional ~2.5 kb band, identically amplified from wild-type plants, indicating heteroplastomic status.

Northern blot was carried out to investigate the transcript level of *gfp* and *bFGF*. As shown in Figure 3C, no strong differences of hybridization signal intensity was observed between *gfp* and *bFGF* genes, indicating the similar accumulation of mRNAs before translation into proteins unless the hybridizations are obviously probe sequence dependent.

Western blot revealed proteins of approximately 17 kDa in lanes 1 to 4, representing bFGF expression in the four T_0_ transgenic lines (Figure 3D) of identical size to commercially produced bFGF. As expected, no signal was observed for wild-type plant.

### 2.4. Measurement of bFGF and GFP Expression in T_0_ Transformed Plants by ELISA

**Figure 4:**
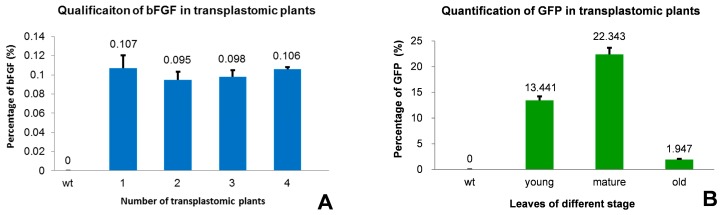
Quantification of bFGF and GFP in transgenic tobacco plants. (**A**) Level of bFGF expression in the leaves of four independent transgenic plants was shown in relation to total soluble protein (mean ± SD). 1–4, independent transplastomic plants; wt, Wild-type plant; (**B**) Level of GFP expression in young, mature and old leaves of transplastomic plants; wt, Wild-type plant. All measurements were made in triplicate.

Total soluble protein (TSP) was extracted from the leaves of T_0_ transformed plants to quantify the expression of bFGF and GFP by ELISA. In the four transgenic plants bFGF represented between 0.095% and 0.106% of TSP from mature leaves (Figure 4A) and much lower expression in young and old leaves (data not shown), indicating a far lower level of bFGF expression than GFP, which the maximum average expression level can reach up to 22.343% of TSP in mature leaves. The average expression in young and old leaves is 13.441% and 1.947% of TSP, relatively lower than mature leaves (Figure 4B).

## 3. Discussion

Plant-made pharmaceuticals (PMPs), both nuclear and plastid stable transformation, offer several unique advantages over microorganism and mammalian systems, allowing production in large scales but low cost and lack of endotoxins and pyrogens. This had been recognized as a promising potential method for the production of therapeutic proteins [28,29]. Some plants like peas, which have high protein content and excellent storage capacity, are an ideal platform for production of pharmaceuticals [30]. Although no plastid-produced products have been used in clinical trials yet, vaccines produced by nuclear transformation of plants or TMV-based transient expression in plants have been tested in the clinic [23,29].

bFGF has previously been expressed by nuclear transformation system and the yield, 2.3% TSP in soybean seeds and 9.55% TSP in rice seeds [15,16], is rather higher than the production in the present experiment, but considering that: (1) the biomass of tobacco mature leaves with average yield of 2.7 tons per hectare [31]; (2) the tobacco needs approximately 40–50 days from seed to fully-expanded leaves; and (3) it is practicable to harvest five to seven times annually under field condition depending on the growing season, the bFGF productivity of transgenic tobacco might be more competitive compared with soybean and rice. The homoplasmic expression of bFGF was achieved in this study, but accumulation needs to be enhanced greatly (Figure 4A). To do this, some possible measures could be taken including N-terminus and tag-added modification [32]. In addition, chloroplast-expressed proteins are generally soluble [22], making this system more attractive for solving the insolubility of protein expressed by conventional nuclear transformation [16]. This study can also pave the way for bFGF expression in other plants, especially vegetables which is feasible for oral delivery without fermentation, purification, cold storage and transportation [22].

Another puzzling question is the expression of GFP which located in the same cassette with bFGF. GFP expression demonstrated far higher level (Figure 4B) than bFGF. Since all of the possible parameters were considered to maintain the uniformity in Northern hybridization operation except the length of two probe sequences, the similar signal intensity should reflect the amount of mRNA (Figure 3C). The strong disparity in accumulation of two proteins therefore could not be explained by their respective mRNA stability alone. It is reported that protein accumulation in transplastomic plants was very likely controlled by post-translational regulation at the level of protein stability [33,34,35] and three factors were experimentally recognized to affect their stability, including the penultimate N-terminal amino acid residue, an N-end rule-like protein degradation pathway and additional sequence determinants in the N-terminal region as well [32]. In our study, the different production of bFGF and GFP is somehow caused by protein degradation as there is no detectable difference was visible at least in transcriptional level. We also cannot exclude the possibility of other factors. For example, the initiation of translation might be affected strongly by 3’ UTRs [36] and therefore the 3’UTR in this study, the leader sequences of *g10* and *clpP* genes, might contribute the gap of translation initiation efficiency to a certain extent [37]. In addition, the small-molecule bFGF, unlike well-known stable GFP, is reported to be unstable in the presence of many proteases [38]. To improve the stability of recombinant protein, the small ubiquitin-related modifier fusion could be superior to glutathione *S*-transferase in enhancement of the expression and solubility of difficult-to-express proteins [39]. According to the proposed hypothesis [32], future attempts to express bFGF in tobacco plastids may employ artificially optimized tag(s) to promote the solubility and stability of the products with less energy consumption of the host cells. 

## 4. Materials and Methods

### 4.1. Plant Materials

Tobacco (*Nicotiana tabacum* cv. Petit Havana) seeds were surface-sterilized and grown on half-strength MS medium supplemented with 8 g/L agar and 30 g/L sucrose with a 16-h light/8-h dark photoperiod. Young leaves were used for plastid DNA extraction and transformation.

### 4.2. Vector Construction for Chloroplast Transformation

According to the complete tobacco chloroplast genome sequences (accession number: NC-001879), two primers (P1: ACAGAGGATGCAAGCGTTAT and P2: CACTGAGCGATCATTTAGGG) were designed to amplify flanking sequences, representing the 16S-trnI-trnA-23S region of the tobacco plastome, for vector construction. PCR was carried out with 30 cycles of 45 s at 95 °C, 40 s at 50 °C, and 2 min at 72 °C, with a 5 min pre-denaturation before the first cycle at 95 °C and a 10 min final extension at 72 °C. The PCR product was inserted into vector pEASY Blunt Simple (Transgen Biotech Co., Ltd., Beijing, China) and verified by sequencing to generate pEASY-Nt. An expression cassette was obtained, as previously described [25]. The whole cassette was amplified by PCR and the product was inserted into the pEASY-Nt vector to form a new vector named pEASY-synth. 

Codon optimization of the *bFGF* encoding target gene was carried out using the software Synthetic Gene Designer (http://www.evolvingcode.net/codon/sgd/index.php). The 474bp optimized *bFGF* gene was inserted into a multiple cloning site (MCS) to yield the final expression vector pXWL-Nt03. (Figure 1). Sequencing revealed that no errors had been introduced.

### 4.3. Chloroplast Transformation and Homoplastomic Selection

Tobacco chloroplast transformation was carried out according to the protocol previously described [40]. Briefly, young leaves of *in vitro* cultured tobacco plants were bombarded using PDS 1000/He biolistic particle delivery system (Bio-Rad, Hercules, CA, USA) with 50 mg of 0.6 µm gold particles coated with 10 µg of plasmid DNA at 1100 psi. Putative transformants were regenerated in MS medium containing 500 mg/L spectinomycin at the first round of selection. The PCR-confirmed leaves of resistant shoots were cut into pieces and subjected to three additional regeneration cycles in selective medium. T_1_ seeds were harvested from T_0_ homoplastomic plants proved by molecular identification and sowed the seeds in pots until to T_3_ generation.

### 4.4. Molecular Testing

Total genomic DNA was extracted from fully expanded young leaves of both transplastomic plants and untransformed wild-type plants using the cetyltrimethylammonium bromide (CTAB) protocol [41]. PCR was carried out under the following conditions: 95 °C for 45 s, 50 °C for 40 s, 72 °C for 2 min; 35 cycles with a 5 min pre-denaturation before the first cycle at 95 °C and a 10 min final extension at 72 °C. The sequence of primer pair designed to check expression cassette was Ec1 (GGTCGGAACAAGTTGATAG) and Ec2 (CAGTAGAGTCTTTCAGTGGC), respectively. The expected PCR band will be ~3.4 kb due to insertion of the foreign expression cassette.

Integration of the foreign expression cassette into the tobacco plastid genome was assessed by Southern blot. The total DNA extracted from young leaves was digested with *Bam*H I and *Kpn* I (New England Biolabs, Hitchin, UK) overnight at 37 °C and subjected to 0.8% agarose gel electrophoresis, then transferred onto positive-charged Hybond-N^+^ nylon membrane (Roche, Basel, Switzerland). Cross-link twice for 30 s each time with 3 min interval. Pre-hybridization and hybridization were carried with the DIG High Prime DNA Labeling and Detection Starter Kit II (Roche) according to the manufacturer’s instructions. The DNA-fixed membrane was hybridized at 42 °C for 16 h with the probe in a hybridization oven before washing and visualization. 

Total RNA was prepared using RNAiso Reagent (TaKaRa Co., Ltd., Dalian, China) and the manufacturer’s instruction from four transgenic lines to test the mRNA transcriptional level by Northern blotting analysis. The *gfp* and *bFGF* genes were amplified as probes. An amount of 5 μg of RNA was loaded in each well and fractionated on a 2.0% denaturing agarose gel, and then transferred onto a nylon membrane for cross-linking. Labeled two probes were hybridized at 65 °C for 16 h in a hybridization oven. Membrane was washed and visualized accordingly. 

### 4.5. Western Blot Assay

Expression of bFGF was measured by Western blot. Briefly, crude protein extracts were extracted from PCR-positive plants, separated by a 15% SDS-PAGE gel, then transferred to a nitrocellulose membrane (0.22 μm, Millipore, Darmstadt, Germany) using a trans-blot semi-dry electrophoretic transfer cell (200 mA, 30 min, Bio-Rad). The nitrocellulose membrane was then saturated with 5% bovine serum albumin (BSA) (*w*/*v*) in TBS-Tween 20 [25 mM Tris-HCl pH 7.6, 0.15 M NaCl, 0.05% (w/v) Tween-20] for 1 h. The membrane was incubated with bFGF-directed antibody (1:100, Santa Cruz Biotechnology Inc., Dallas, TX, USA), prior to incubation with the secondary antibody (1:3000, goat anti-mouse IgG alkaline phosphatase conjugate, Novagen, Madison, WI, USA). Labeled proteins were visualized by adding a substrate BCIP/NBT (Roche).

### 4.6. ELISA

To measure the expression level of recombinant protein in transplastomic plants, total soluble protein was extracted from fully expanded leaves of both transformed and wild-type plants. The leaves were cut into pieces and grounded by liquid nitrogen, homogenized in PBS buffer (137 mM NaCl, 2.7 mM KCl, 10 mM Na_2_HPO_4_, 2 mM KH_2_PO4). ELISA was performed using commercial anti-bFGF antibodies, and recombinant bFGF as standard (Sigma, St. Louis, MO, USA), as previously described [42]. Similarly, GFP expression was measured in young, mature and old leaves individually, and analyzed the data statistically. Wild-type plant was used as control.
